# Can Artificial Intelligence Diagnose Knee Osteoarthritis?

**DOI:** 10.2196/67481

**Published:** 2025-04-23

**Authors:** Mihir Tandon, Nitin Chetla, Adarsh Mallepally, Botan Zebari, Sai Samayamanthula, Jonathan Silva, Swapna Vaja, John Chen, Matthew Cullen, Kunal Sukhija

**Affiliations:** 1 Albany Medical College Albany, NY United States; 2 University of Virginia School of Medicine Charlottesville, VA United States; 3 School of Medicine Virginia Commonwealth University Richmond, VA United States; 4 St. James School of Medicine Binghamton, NY United States; 5 Rush Medical College Chicago, IL United States; 6 Kaweah Health Visalia, CA United States

**Keywords:** large language model, ChatGPT, GPT-4o, radiology, osteoarthritis, machine learning, X-rays, osteoarthritis detection

## Abstract

This study analyzed the capability of GPT-4o to properly identify knee osteoarthritis and found that the model had good sensitivity but poor specificity in identifying knee osteoarthritis; patients and clinicians should practice caution when using GPT-4o for image analysis in knee osteoarthritis.

## Introduction

Osteoarthritis often affects the knee, causing pain and disability, and is typically diagnosed by X-ray [1]. Advancements in artificial intelligence (AI) offer potential to automate image analysis, reducing diagnostic burden [2]. Given its widespread availability, tools like ChatGPT have potential as point-of-care diagnostic aids. AI has already been incorporated on the physician side through clinical decision support systems and robotic surgery. On the patient side, AI is used in applications such as virtual health assistants [3].

Orthopedic surgeons, radiologists, and primary care physicians can use AI tools to streamline their workflows and reduce errors while analyzing imaging for pathologies like osteoarthritis. Moreover, patients use ChatGPT to analyze their imaging to further understand their condition [4]. The ability of AI to read other radiological images (eg, computed tomography angiograms) has been shown to be subpar [5]. However, studies have shown that AI can perform well with X-rays [6]. As such, it is increasingly important for physicians to understand AI’s strengths and limitations to assess its use in imaging and guide patients using AI for self-diagnosis.

## Methods

We queried ChatGPT (using the GPT-4o version) and assessed its performance in classifying 500 X-ray images of normal knees and 500 images of knees with osteoarthritis from a publicly available Kaggle database [7]. Images were verified based on consensus among radiologists. A single standardized prompt was used: “This is an x-ray image found on examination, the multiple-choice question is as follows. Based on the x-ray image, does the patient have A) no osteoarthritis, B) osteoarthritis.” Key metrics included accuracy, sensitivity, and specificity. No images were rejected by ChatGPT. The code used for statistical analysis is included in Multimedia Appendix 1.

## Results

**Figure 1:**
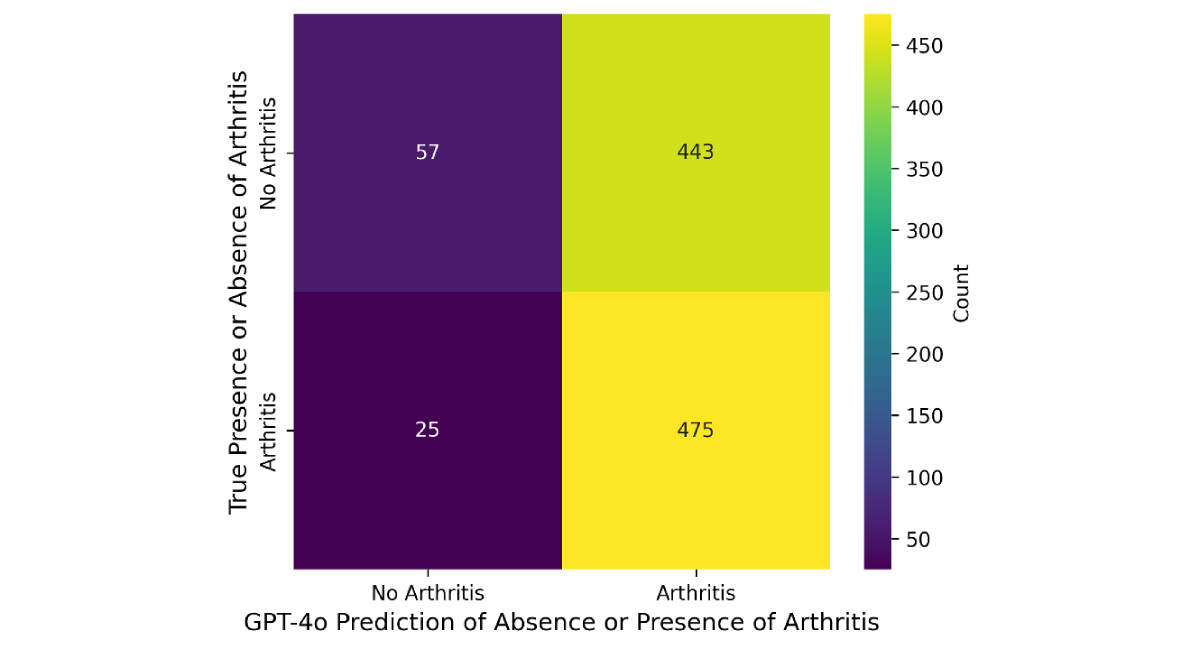
Sensitivity and specificity of Chat-GPT4o in analyzing knee osteoarthritis X-rays.

The model’s performance in distinguishing osteoarthritis from nonosteoarthritis knee X-rays was mixed. The high recall (0.950, 95% CI 0.964-0.943) suggests that the model was sensitive in identifying arthritis cases, while the low specificity (0.114, 95% CI 0.134-0.104) indicated a poor ability to correctly identify nonosteoarthritis cases. The *F*_1_-score (0.670, 95% CI 0.699-0.655) balanced precision and recall, showing moderate effectiveness, but the precision (0.517, 95% CI 0.548-0.501) reflected that about half the predicted osteoarthritis cases were correct. Accuracy was 0.532 (95% CI 0.563-0.516). Figure 1 shows sensitivity and specificity.

The binomial test, where the null hypothesis assumed the model’s accuracy was 50% or less, indicated that the model was statistically better than random chance (*P*=.02). Additionally, the *χ*^2^ test (*P*<.001) indicated a strong dependence between the model’s predictions and the actual labels, demonstrating that its classifications were not purely random. However, the significance of this test should be interpreted with caution, as it does not necessarily reflect high accuracy or clinical reliability.

## Discussion

The model had difficulty distinguishing between “not arthritis” and “arthritis.” While the recall for arthritis was high (0.950), indicating strong performance in identifying true arthritis cases, the low specificity (0.114) reflects a significant number of false positives, with many nonarthritis cases misclassified as arthritis. This bias toward predicting arthritis lowered precision (0.517) and accuracy (0.532); similar misclassification issues have been reported in other ChatGPT studies [8].

Limitations include, first, that the prompt was binary. A binary prompt was used because it would have been difficult to analyze data obtained with an open-ended prompt. Second, the dataset was small; a larger dataset would have yielded more robust conclusions.

Even with its limitations, this study presents important data on GPT4o’s use in imaging for diagnosing osteoarthritis. This is vital, as our understanding of tools like this in health care contexts is limited. These results suggest a need for better class balance and improved feature differentiation. Similar misclassification patterns have been noted in previous studies, where overlapping features led to false positives [9]. A higher-resolution, more comprehensively annotated osteoarthritis dataset could improve model training, enhancing overall accuracy, sensitivity, and specificity. Thus, future work should focus on analyzing larger datasets and refining the model to handle more nuanced cases more effectively, improving performance statistics. Using image preprocessing techniques, such as contrast enhancement and noise reduction, and including metadata like medical history and clinical presentation could also help distinguish osteoarthritis from anatomical variations.

Our results suggest that clinicians should use ChatGPT cautiously and as a screening tool prior to their own validation to help mitigate misclassification. Clinicians should also educate patients about the risks of using AI for self-diagnosis of osteoarthritis based on X-rays. Despite its shortcomings, AI has potential for developing more reliable diagnostic models for osteoarthritis.

## Appendix

Multimedia Appendix 1 Code for analysis and prompting.

## Abbreviations

AI: artificial intelligence
