# Correction: Can Artificial Intelligence Diagnose Knee Osteoarthritis?

**DOI:** 10.2196/82980

**Published:** 2025-09-12

**Authors:** Mihir Tandon, Nitin Chetla, Adarsh Mallepally, Botan Zebari, Sai Samayamanthula, Jonathan Silva, Swapna Vaja, John Chen, Matthew Cullen, Kunal Sukhija

**Affiliations:** 1 Albany Medical College Albany, NY United States; 2 University of Virginia School of Medicine Charlottesville, VA United States; 3 School of Medicine, Virginia Commonwealth University Richmond, VA United States; 4 St. James School of Medicine Binghamton, NY United States; 5 Rush Medical College Chicago, IL United States; 6 Kaweah Health Visalia, CA United States

In “Can Artificial Intelligence Diagnose Knee Osteoarthritis?” (JMIR Biomed Eng 2025;10:e67481), the authors made two corrections.

In the originally published version, Figure 1 displayed two of the Y-axis labels incorrectly. The label *“Arthritis”* was placed next to the row representing X-rays without arthritis, and the label *“No Arthritis”* was placed next to the row representing X-rays with arthritis.

**Figure 1:**
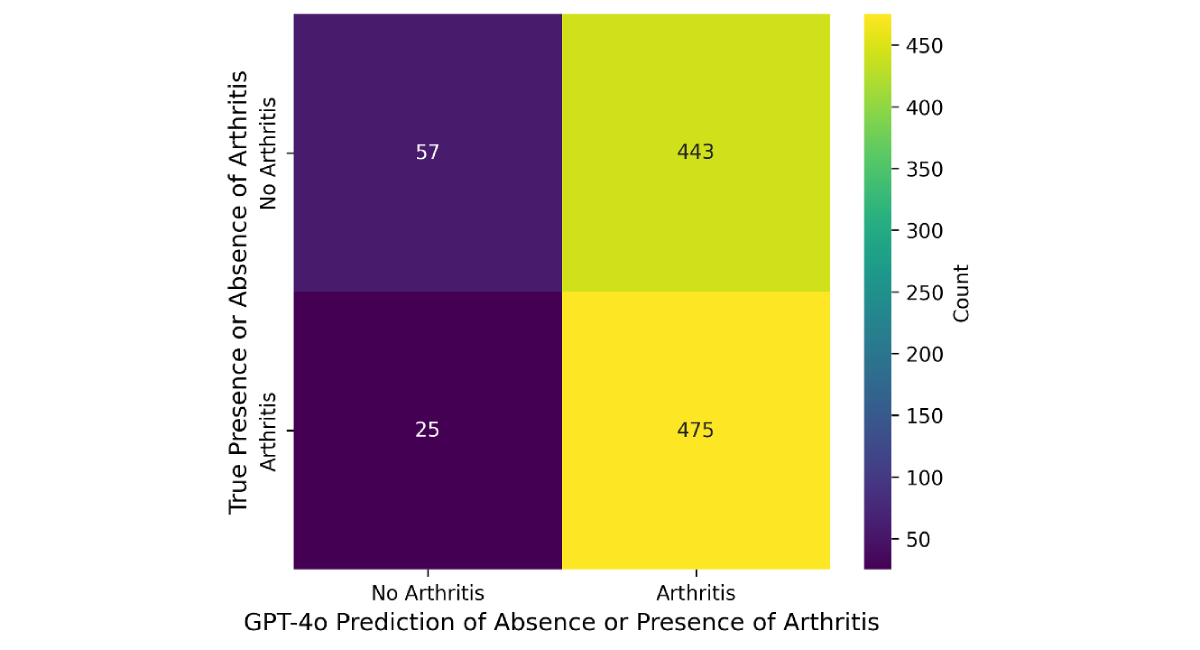
Sensitivity and specificity of Chat-GPT4o in analyzing knee osteoarthritis X-rays.

The text in Figure 1 has been corrected so that the Y-axis labels align with the data:

The top row is labeled *“No Arthritis”*, representing X-rays of knees without arthritis.
The bottom row is labeled *“Arthritis”*, representing X-rays of knees with arthritis.

The X-axis label, *“GPT-4o Prediction of Absence or Presence of Arthritis”* and the Y-axis label, *“True Presence or Absence of Arthritis”*, have been reformatted to span the entire length of the figure rather than being stacked to improve both readability and overall appearance.

Additionally, an **Authors’ Contributions** section has been added to the manuscript using the CREdiT taxonomy format:

Conceptualization: NC (lead), MT (equal), KS (equal)

Data curation: AM (lead), MT (equal), SS (supporting), SV (supporting), JC (supporting)

Formal analysis: JC (lead), JS (supporting), MC (supporting), SV (supporting), AM (supporting)

Funding acquisition: KS (lead)

Investigation: SS (lead), KS (equal), BZ (supporting), SV (supporting)

Methodology: MT (lead), NC (equal), KS (equal), AM (supporting)

Resources: SV (lead), JC (supporting)

Software: JC (lead), AM (supporting)

Supervision: KS (lead), MT (equal), NC (equal)

Validation: JS (lead), JC (equal), MC (equal)

Visualization: MT (lead), MC (equal), SS (supporting)

Writing – original draft: MT (lead), NC (equal), BZ (supporting), SS (supporting), AM (supporting)

Writing – review & editing: JS (lead), SV (equal), JC (equal), MC (supporting), KS (supporting)

The correction will appear in the online version of the paper on the JMIR Publications website together with the publication of this correction notice. Because this was made after submission to PubMed, PubMed Central, and other full-text repositories, the corrected article has also been resubmitted to those repositories.

