# Supplementary material for: Can Artificial Intelligence Diagnose Knee Osteoarthritis?
Source: JMIR Biomed Eng. 2025 Apr 23;10:e67481. doi: 10.2196/67481 (PMC12059495; doi:10.2196/67481)
Supplement: Multimedia Appendix 1 [file biomedeng_v10i1e67481_app1.docx]

**Appendix 1: Code for Analysis and Prompting**

**Code for Querying ChatGPT**

import base64

import requests

import os

import pandas as pd

api_key = “” # insert own api key here

# Function to encode the image to base64

def encode_image(image_path):

  with open(image_path, "rb") as image_file:

    return base64.b64encode(image_file.read()).decode('utf-8')

# Replace 'path_to_your_image.jpg' with the path to your actual image file

# images path

image_file_path = os.fsencode(r"C:/Users/Jo/Documents/nitin_chatgpt_project/arthiritis_project/train/train/Osteoarthritis")

# empty list of dictionaries

data = []

# run for first x files in the directory

for file in os.listdir(image_file_path)[:500]:

    # print(os.fsdecode(file))

    # filename = os.fsdecode(file)

    dict = {}

    # TODO: check if file is in the list of files in bcc_scc_dataframe.csv

    if ('.png' in file.decode('ascii')):

        base64_image = encode_image(os.path.join(image_file_path, file))

        # base64_image = encode_image(image_path)

        headers = {

          "Content-Type": "application/json",

          "Authorization": f"Bearer {api_key}"

        }

        payload = {

          "model": "gpt-4o",

          "messages": [

            {

              "role": "user",

              "content": [

                {"type": "text", "text": '''This is an image found on examination, the multiple choice question is as follows.

                Based on the image, does the patient have A) no Osteoarthritis, B) Osteoarthritis. Only output the answer as A or B.'''},

                {

                  "type": "image_url",

                  "image_url": {

                    "url": f"data:image/jpeg;base64,{base64_image}"

                  }

                }

              ]

            }

          ],

          "max_tokens": 300

        }

        # Make the API request and print out the response

        response = requests.post("https://api.openai.com/v1/chat/completions", headers=headers, json=payload)

        # parse out chatgpt message that we care about: add filename and the corresponding chatgpt response to dataframe

        try:

            print(file.decode('ascii'), ": ", response.json().get('choices')[0].get('message').get('content'))

            dict['filename'] = file.decode('ascii')

            dict['result'] = response.json().get('choices')[0].get('message').get('content')

            data.append(dict)

        # got an empty response rarely

        except TypeError:

            print(file.decode('ascii'), ": ", "")

            dict['filename'] = file.decode('ascii')

            dict['result'] = ""

            data.append(dict)

        # for anything else that I didn't expect

        except:

            print("Unresolved error: excluding the data")

# write all responses to a file

final_data = pd.DataFrame(data)

final_data.to_csv("C:/Users/Jo/Documents/nitin_chatgpt_project/arthiritis_project/arthritis.csv", sep=',', index=False, encoding='utf-8')

**Code for Generating Statistics**

import pandas as pd

import seaborn

import numpy as np

import matplotlib.pyplot as plt

from sklearn import metrics

import scipy.stats

data_melanoma = pd.read_csv('arthritis.csv')

data_normal = pd.read_csv('no_arthritis.csv')

data_melanoma = data_melanoma.assign(truth = np.ones(len(data_melanoma['filename'])))

data_normal = data_normal.assign(truth = np.zeros(len(data_normal['filename'])))

data_list = [data_melanoma, data_normal]

data = pd.concat(data_list, ignore_index=True)

data['result'].replace(['B.','A.'], [1,0], inplace=True)

data['result'].replace(['B','A'], [1,0], inplace=True)

data['result'].replace(['B)','A)'], [1,0], inplace=True)

data = data.drop(['filename'], axis=1)

for x in data['result']:

    if x != 0 and x != 1:

        data['result'].replace([x], [None], inplace=True)

data = data.dropna()

data = data.astype(int)

for x in data['truth']:

    if type(x) != int:

        print(x)

confusion_matrix = metrics.confusion_matrix(data['truth'], data['result'])

cm_display = metrics.ConfusionMatrixDisplay(confusion_matrix = confusion_matrix, display_labels = ["not arthritis", "arthritis"])

cm_display.plot()

plt.show()

F1 = metrics.f1_score(data['truth'],data['result'], average='binary')

recall = metrics.recall_score(data['truth'],data['result'], average='binary')

precision = metrics.precision_score(data['truth'],data['result'], average='binary')

accuracy = metrics.accuracy_score(data['truth'],data['result'])

print("Accuracy: ", accuracy)

print("F1: ", F1)

print("Recall: ", recall)

print("Precision: ", precision)

result = scipy.stats.binom_test(475+57, n=1000, p=0.5, alternative='greater')

result

#chi2 = scipy.stats.chisquare([82, 918], f_exp=[500, 500])

chi2, p_value, dof, expected = scipy.stats.chi2_contingency(confusion_matrix)

p_value

*Note: Codes were created on Jupyter Notebook, so it would not work properly if the code is just copy pasted into a .py file*
